# Editorial: Point-of-care testing for infectious and foodborne pathogens

**DOI:** 10.3389/fcimb.2022.975449

**Published:** 2022-07-28

**Authors:** Hongchao Gou, Jianmin Zhang, Ming Liao

**Affiliations:** ^1^ Institute of Animal Health, Guangdong Academy of Agricultural Sciences, Guangzhou, China; Guangdong Provincial Key Laboratory of Livestock Disease Prevention, Guangzhou, China; Maoming Branch, Guangdong Laboratory for Lingnan Modern Agriculture, Maoming, China; Scientific Observation and Experiment Station of Veterinary Drugs and Diagnostic Techniques of Guangdong Province, Guangzhou, China; ^2^ National and Regional Joint Engineering Laboratory for Medicament of Zoonoses Prevention and Control; Key Laboratory of Zoonoses, Ministry of Agriculture; Key Laboratory of Zoonoses Prevention and Control of Guangdong Province; Key Laboratory of Animal Vaccine Development, Ministry of Agriculture; Guangdong Laboratory for Lingnan Modern Agriculture; College of Veterinary Medicine, South China Agricultural University, Guangzhou, China

**Keywords:** infectious and foodborne pathogens, coronavirus disease 2019 (COVID-19), point-of-care testing (POCT), isothermal amplification, multiplex real-time PCR

Infectious and foodborne pathogens usually pose emergency threat to the human and animal health. In recent years, they are gaining more attention due to emerging and re-emerging outbreaks, such as Influenza virus, Severe Acute Respiratory Syndromes virus, Ebola virus, Norovirus, *Salmonella Typhimurium*, *Escherichia coli* O157, Hepatitis E Virus, African swine fever virus and so on. Since December 2019, the outbreak of coronavirus disease 2019 (COVID-19) around the world was declared as a disease of “public health emergency of international concern” by the World Health Organization (WHO).

Rapid and early detection of infectious and foodborne pathogens display a dramatic impact in controlling and preventing an outbreak. To face current challenges regarding infectious and foodborne pathogens, a point-of-care testing (POCT) concept has been introduced to detection technologies and devices. Especially for COVID-19, POCT technologies displayed the advance of user-friendly, rapid detection, so they can be directly used on-site or at home. This Research Topic is focused on novel ideas in POCT technologies and devices for infectious and foodborne pathogens, aimed at improving on-site application of rapid diagnostic techniques by detecting analytes including antigens, nucleic acids and specific antibodies for microorganisms.

Of great concern to the increasing incidence of acute respiratory tract infections (RTI), which leads to high mortality in children and adults worldwide in recent years, Gradisteanu Pircalabioru et al. reviewed the advances in microbiological diagnostic of viral RTI in this Research Topic. They provided a non-exhaustive overview of conventional viral detection and infection monitoring methods and technological improvements. Focused on miniaturized systems and evaluating the clinical perspectives for further use as POCT, they discussed the potential of immunoassays and nucleic acid (NA) amplification and the new approaches such as microfluidics and biosensors-based techniques as rapid diagnostic platforms for viral respiratory infections detection methods and monitoring. Since viral infections impose stringent detection and spread monitoring, they presented the emerging Internet-of-Things (IoT) and highlight their potential as a future solution in the virology diagnostic and respiratory infections prophylaxis.

In the face of the sudden outbreak of COVID-19, Daoud et al. validated two commercial kits for the detection of IgM and IgG using lateral flow immunoassay tests and to study the effect of the combination of both serology kits for better detection of immunoglobulins. The results showed sensitivities for IgM detection varying between 58.9 and 66.2% for the kits alone and 87.7% of the combination of both kits. IgG detection was not significantly affected by this combination. Both kits manifested high specificities (99.2–100%). Chen et al. developed a novel molecular diagnosis technique, named multiplex reverse transcription loop-mediated isothermal amplification linked to a nanoparticle-based lateral flow biosensor (mRT-LAMP-LFB). This test was applied to detect SARS-CoV-2 based on the SARS-CoV-2 RdRp and N genes. The full process, including reaction preparation, viral RNA extraction, RT-LAMP, and product identification, could be achieved in 80 min. The mRT-LAMP-LFB detection results were consistent with the Real-Time RT-PCR Kit (Sansure biotech Inc, China) in the evaluation of clinical samples. To identify SARS-CoV-2 variants, Niu et al. established a highly sensitive and portable on-site detection method for the HV69-70del which exist in SARS-CoV-2 Alpha and Omicron variants using a PCR-based CRISPR/Cas13a detection system (PCR-CRISPR). The results showed that the PCR-CRISPR detection method can detect 1 copies/μL SARS-CoV-2 HV69-70del mutant RNA and identify 0.1% of mutant RNA in mixed samples, which was more sensitive than the RT-qPCR based commercial SARS-CoV-2 variants detection kits and sanger sequencing. Additionally, by combining PCR-CRISPR with lateral flow strip, they provided a novel diagnosis tool to identify SARS-CoV-2 variants in primary and resource-limited medical institutions without professional and expensive fluorescent detector.

Hepatitis C virus (HCV) infection is a global public health threat. While immunoassays and qPCR play a significant role in detecting HCV, rapid and accurate point-of-care testing is important for pathogen identification. Wang et al. established a reverse transcription recombinase-aided amplification-lateral flow dipstick (RT-RAA-LFD) assay to detect HCV. Using extracted RNAs from 46 anti-HCV antibody-positive samples, RT-RAA-LFD showed 100% positive and negative concordance rates with qPCR. The RT-RAA-LFD assay established is suitable for the rapid clinical detection of HCV at the community level and in remote areas.

African swine fever (ASF) is a highly contagious and usually deadly porcine infectious disease listed as a notifiable disease by the World Organization for Animal Health (OIE). A sensitive, specific, rapid, and simple molecular point of care testing for African swine fever virus (ASFV) B646L gene in blood samples was established by Zhang et al., including treatment of blood samples with simple dilution and boiling for 5 min, isothermal amplification with recombinase-aided amplification (RAA), and visual readout with lateral flow assay (LFA) at room temperature. Without the need to extract viral DNA in blood samples, the intact workflow from sampling to final diagnostic decision can be completed with minimal equipment requirement in 30 min. Evaluation of clinical blood samples of RAA-LFA showed 100% coincident rate with OIE-recommended PCR, in testing both extracted DNAs and treated bloods. They also found that some components in blood samples greatly inhibited PCR performance, but had little effect on RAA. Inhibitory effect can be eliminated when blood was diluted at least 32-64-fold for direct PCR, while only a 2-4 fold dilution of blood was suitable for direct RAA, indicating RAA is a better choice than PCR when blood was used as detecting sample. Wang et al. established a cleaved probe-based loop-mediated isothermal amplification (CP-LAMP) detection method for ASFV. Based on the original primer sets, they targeted the ASFV 9GL gene sequence to design a probe harboring a ribonucleotide insertion. Ribonuclease H2 (RNase H2) enzyme activity can only be activated when the probe is perfectly complementary, resulting in hydrolytic release of a quencher moiety, and consequent signal amplification. Visualization of the fluorescence product was employed using a self-designed 3D-printed visualization function cassette, and the CP-LAMP method achieved specific identification and visual detection of ASFV. Porcine parvovirus (PPV) is an important cause of pig reproductive diseases. A rapid, visible, and economical clinical diagnostic strategy to detect PPV is necessary. By using three pairs of crRNA primers targeted to the VP2 gene, an ERA-CRISPR/Cas12a system for PPV detection was successfully developed by Wei et al. The approach involved isothermal detection at 37°C, and the method can be used for visual inspection.

For the rapid detection of foodborne pathogens, isothermal real-time recombinase polymerase amplification (RPA) and lateral flow strip detection (LFS RPA) were used. The LFS RPA targeted to the conserved sequence of invasion protein A (invA) to detect Salmonella spp was employed by Zhao et al. To quickly and directly detetct *Mycoplasma bovis* (*M. bovis*) in bovine milk, an RPA assay based on the fluorescence monitoring (real-time RPA) and an RPA assay combined with a lateral flow strip (LFS RPA) were conducted by Li et al. Lu et al. developed a Cas12a-assisted rapid isothermal detection (CARID) system for the detection of toxigenic *V. cholerae* serogroups O1 and O139 by combining recombinase-aided amplification and CRISPR-Cas (clustered regularly interspaced short palindromic repeats and CRISPR-associated proteins). The results can be determined by fluorescence signal and visualized by lateral flow dipstick. Multiple-CARID was also established for efficiency and economic considerations with an acceptable decrease in sensitivity. Simulated sample tests showed that CARID was suitable for complex samples. The conventional serotyping methods for differentiating *Salmonella* serovars are complicated, time-consuming, laborious, and expensive; therefore, rapid and accurate molecular diagnostic methods are needed for effective detection and prevention of contamination. Xin et al. developed and evaluated a TaqMan multiplex real-time PCR assay for simultaneous detection and differentiation of the *S. Pullorum*, *S. Gallinarum*, *S. Enteritidis*, and *S. Typhimurium*. It achieved comparable results to the traditional bacteriological examination. Meanwhile, a multiplex TaqMan-based real-time PCR assay was developed on the BD MAX platform by Li et al. This assay can simultaneously detect and differentiate *V. cholerae* and *V. parahaemolyticus* directly from human fecal specimens. The BD MAX assay was evaluated for its performance compared with conventional real-time PCR after automated DNA extraction steps, using 164 retrospective stool samples. The overall percent agreement between the BD MAX assay and real-time PCR was ≥ 98.8%.

In this Research Topic, some equipment also showed an advantage on POCT. For example, the Cepheid GeneXpert^®^ (Xpert) CT/NG assay can be performed on the GeneXpert instrument platform in laboratories and is simple to operate. Han et al. reported that the Xpert CT/NG test exhibited high sensitivity and specificity in the detection of *Chlamydia trachomatis* (CT) and *Neisseria gonorrhoeae* (NG) in both urine and cervical samples when compared to the reference results. The 90-min turnaround time for CT and NG detection at the point of care using Xpert may enable patients to receive treatment promptly. Because the emergence and spread of the novel mobile Tet(X) tetracycline destructases confer high-level tigecycline and eravacycline resistance in *Escherichia coli* and *Acinetobacter* spp. and pose serious threats to human and animal health, a rapid and robust Tet(X) detection assay was urgently needed to monitor the dissemination of tigecycline resistance. Based on matrix-assisted laser desorption ionization-time of flight mass spectrometry (MALDI-TOF MS), Cui et al. developed a rapid and simple assay to detect Tet(X) producers in Gram-negative bacteria. This MALDITet(X) test was based on the inactivation of tigecycline by a Tet(X)-producing strain after a 3-h incubation of bacterial cultures with tigecycline. Culture supernatants were analyzed using MALDI-TOF MS to identify peaks corresponding to tigecycline (586 ± 0.2 m/z) and a tigecycline metabolite (602 ± 0.2 m/z). The results were calculated using the MS ratio [metabolite/(metabolite + tigecycline)]. The sensitivity of the MALDITet(X) test with all 216 test strains was 99.19%, and specificity was 100%. The test can be completed within 3 h.

POCT requires that all of the analytical processes, from sample collection to result communication, should be performable in one or a few simple steps to reduce time and costs between the test and treatment. In general, some detection methods and equipment reported in this Research Topic have yet to be fully applied in POCT. They are likely to be improved by combining with microfluidics, biosensors, wireless cell phone based technologies, paper based devices and other advanced techniques in the future.

## Author contributions

HG drafted the manuscript. JZ and ML participated the revision of this manuscript. All the authors read and approved the final manuscript.

## Funding

This work was supported by grants from the Special fund for scientific innovation strategy-construction of high level Academy of Agriculture Science-Distinguished Scholar (R2020PY-JC001), the Project of Collaborative Innovation Center of GDAAS (XTXM202202), the Scientific innovation strategy-construction of high level Academy of Agriculture Science (202110TD), the Guangzhou Science and Technology Bureau (202103000096 and 202206010192) the Special Fund for Scientific Innovation Strategy-Construction of High Level Academy of Agriculture Science (R2017YJ-YB2005 and R2021PY-QY006) and the Start-up Research Project of Maoming Laboratory (2021TDQD002).

## Conflict of interest

The authors declare that the research was conducted in the absence of any commercial or financial relationships that could be construed as a potential conflict of interest.

## Publisher’s note

All claims expressed in this article are solely those of the authors and do not necessarily represent those of their affiliated organizations, or those of the publisher, the editors and the reviewers. Any product that may be evaluated in this article, or claim that may be made by its manufacturer, is not guaranteed or endorsed by the publisher.

